# Hospital and Institutional News

**Published:** 1917-02-17

**Authors:** 


### HOSPITAL AND INSTITUTIONAL NEWS.

### THE NEW YEAR'S HONOURS.

Broadly speaking, the delayed New Year's Honour List which appeared on February 13 is mainly a War Work List; and institutional workers as such find little place in it. There are, however, a good many names that are known in institutional circles: doubtless because the man who can make his mark in such a sphere is very likely to make it also in anything he sets his hand to. Mr. T. C. Dewey, for instance, best known as the chairman of the Prudential Assurance Company, becomes a baronet. Mr. Dewey has served on the War Office Expenditure Committee, has long been an enthusiastic supporter of the Territorial movement, and is deputy-chairman of the Royal National Pension Fund for Nurses. Dr. R. Armstrong-Jones, who receives a knighthood, is a distinguished alienist, who retired last year from the service of the L.C.C. after many years at the head of the well-known Claybury Asylum administered by that body. Mr. J. L. Otter, the retiring Mayor of Brighton, is also knighted: he has been conspicuous in his keen service in connection with the Indian hospitals established in the town of which he was Mayor. Lord Dartmouth is to be a Knight Commander of the Bath, chiefly for services to the Territorial Force; but it was only on February 12 that his beautiful town house in Charles Street was opened by the Duchess of Connaught as a Red Cross hospital for seventy soldier patients, so he, too, has done work also for the cause of the sick and wounded.

### THE ORDERS OF KNIGHTHOOD.

Dr. Arthur Newsholme likewise becomes a K.C.B. He is, of course, the well-known Medical Officer to the Local Government Board; and this honour was one that was morally bound to come his way sooner or later. A like honour is awarded to Major-General Sir Charles Crutchley, K.C.V.O., the Governor of Chelsea Hospital. Sir Robert Burnet, the well-known physician to the Royal Household and to two or three London hospitals, is appointed K.C.V.O.; and the Commandership of the same Order is awarded to Mr. J. G. Griffiths, Honorary Secretary of King Edward's Hospital Fund for London. The C.M.G. is conferred upon Dr. John Warnock, Director of Lunatic Asylums in Egypt, and upon Dr. C. L. Sansom, Principal Medical Officer of the Federated Malay States. Mr. John Grice, of Melbourne, receives the honour of Knight Bachelor: he is chairman of the Melbourne Hospital, and a well-known philanthropist.

### AN ALTERATION OF THE LAWS OF THE MIDDLESEX HOSPITAL.

Notice has been given by advertisement in the press of a Court of Governors of the Middlesex Hospital on February 22 next, to consider amongst other things an alteration in the laws of the hospital governing the appointment of a chaplain; and to eliminate from the laws any mention of a religious qualification in regard to all appointments, with the exception of the chaplaincy. This proposition, which is set forth in the notice in one continuous sentence, not even divided by the semicolon inserted above, appears on the face of it to contain two distinct suggestions. In regard to the second half, the abolition of religious tests in connection with appointments at a voluntary hospital, there will, we imagine, be little, if any, dissent. It is amazing enough that in the twentieth century such limitations should still be found in the constitution of a hospital; and, however much they may have been a dead-letter in practice, it is well that they should be formally abolished. The former half of the sentence relates, we understand, to an arrangement under which the chaplaincy of the hospital will be carried out by the incumbent and staff of a neighbouring church. The Samaritan Fund's operations, formerly managed by the chaplain, will now be carried out by the Lady Almoner's department.

### PROPOSED LABORATORY FOR NORTH WALES.

A scheme has been drawn up for the founding of a bacteriological laboratory in connection with the University College of North Wales, Bangor, to form part of the science block for which funds are now being sought. A laboratory for the examination of pathological material to facilitate the diagnosis and treatment of disease has been a want long felt by the medical practitioners in North Wales, and the measures recently introduced by the Local Government Board to combat venereal diseases has made more immediately pressing the need of such a provision. A deputation of medical men has conferred with the Senate of the University College of North Wales to discuss the matter, and the proposals of a Joint Committee of the Senate and the medical men formed as the outcome of that conference are now ready for submission to the public. It is estimated that the necessary buildings could be erected and equipped for about £3,500, and that the maintenance of the laboratory and employment of the necessary staff would cost about £1,200 a year. This income would be derived from sanitary authorities, agricultural societies, and the Board of Agriculture in grants and in fees for work done under the Food and Drugs Act and the Fertilisers and Feeding Stuffs Acts, and in fees from medical men and veterinary practitioners for the examination of pathological material and for vaccines prepared from such material. It is expected that the revenue will suffice to meet the expenditure, and that the project will succeed from a financial point of view.

### LORD DEVONPORT AND THE HOSPITALS.

Some of the chief officers of the larger hospitals are, we understand, seeking an interview with Lord Devonport with the object of ascertaining how far, in his opinion, the voluntary restricted consumption of meat, bread and sugar should apply to hospitals. From the figures we have seen there appears to be little difficulty in bringing the allowances of the patients and staff in respect of bread and sugar within the limits. With meat, however, the weekly allowance of two and a-half pounds of uncooked meat will, without a searching revision of diets, prove quite insufficient for the patients. The staff of course can be relied upon loyally to abide by conditions which would apply to them if they lived outside institutions. In respect of patients the only substantial substitute for meat that occurs first to one is fish, but fish is very hard to get, apart from questions of expense. Of course some hospitals, especially those receiving wounded soldiers, have a larger percentage of patients with hearty appetites than others, but everywhere secretaries will be busy examining diet tables and making calculations as to weights. One thing appears certain, and that is, that the hitherto large allowance per day of uncooked meat for each patient in a military hospital will have to be reconsidered.

### MUNIFICENT BEQUESTS TO THE SUNDAY FUND.

We are glad to have authority to announce that the bequest of the late William Herring to the Metropolitan Hospital Sunday Fund amounts to £100,000, not £1,000 as previously reported. This munificent bequest from the brother of the late George Herring, who for some years previous to his death contributed annually from £12,500 to £10,000 a year to the Sunday Fund, and on his death left property which has yielded an income to the Fund averaging £27,243 for the last ten years, is remarkable. Metropolitan hospitals through the Sunday Fund have good cause for gratitude to the late George and William Herring, who had been staunch supporters of this Fund for many years.

### THE BRENTFORD INFIRMARY DISPUTE.

The long-standing dispute between the Guardians of Brentford and their infirmary medical officers has involved the interference of the Local Government Board; and after inspection by an official of that Department, the Right Hon. Walter Long, M.P., has administered what amounts to a direct rebuke to the Guardians. It may be recalled that the trouble originally arose through the Guardians launching out on a scheme of extensive reform, in spite of the absence of their chief medical officer at the war. Whether that official would have advised them to proceed differently, if he had been on the spot, no one can say; but the result of the Guardians' action was that the acting medical superintendent and the senior assistant medical officer of the infirmary resigned their posts. The Guardians appointed to the superintendentship the junior assistant medical officer, who took his medical qualification as recently as 1914; and they found another young doctor, just returned from the war, to accept a post also. Thus two very junior practitioners have been carrying on the work which formerly occupied three doctors, two of them highly experienced. The Local Government Board's inspector has catalogued the work that ought to be done by these two medical officers, and he says plainly that it cannot possibly be properly done by less than three resident medical officers, one of whom should be of mature experience. In a covering letter the President of the Local Government Board endorses this view, and advises the Guardians to fall in with it. So far the Guardians appear to be contumacious; but we imagine that on second thoughts they will see the futility of quarrelling with the Local Government Board, and will retrace their steps.

### ANOTHER BENEFACTION FOR THE WELSH MEDICAL SCHOOL

It is announced that Dr. William Price, of Southerndown and Park Place, Cardiff, who died a few weeks ago, has bequeathed a handsome sum to the Welsh National Medical School. The gift is in the form of a residue, and though the exact amount is not yet ascertainable, it is estimated that it will be somewhere about £20,000. The Welsh Medical School does not lack friends, and this bequest is but another instance of the loyal generosity it has called forth from the sons of Wales in every walk of life.

### A BIG EXTENSION IN IRELAND.

With the opening of the Abercorn wing of the Ulster Volunteer Force Military Hospital, Belfast, special accommodation has been gained for orthopædic cases, and four wards with 266 beds have been added to the hospital. Sir Robert Liddell, who has been much concerned in the extension, has had one of the wards named after him. About 5 per cent. of the cases received during the past year have required orthopædic treatment, and more than half of these have been fitted with limbs at the hospital of a standard said to compare favourably with the War Office pattern. These limbs are manufactured at Belfast.

### A RED CROSS HERRING.

The editor of the *Spectator* has been hauled over the coals by a correspondent for declaring, presumably in reference to the Red Cross Sale, that "the money of dealers is not likely to reach" the Society "except through the purchase of gifts in kind to the Red Cross." In defending the patriotism and generosity of dealers, Mr. S. H. Whitbread shrewdly points out that the man who can buy and sell a valuable work of art is not necessarily a man with a permanent surplus income. Mr. Spectator meets the charge of innuendo with, it must be confessed, a lame defence. While denying any slur on dealers, he adds that "in these hard times they have to earn money on their capital, and that the only way they can do this is by 'transactions' in promising pictures and curios." Money has to be earned on capital whether times are hard or not, and an art dealer, strange as it may seem, has no simpler way of doing this than by transactions in promising pictures and curios. That is his trade. But let us return to commonsense and admit that the whole question whether dealers subscribe to the Red Cross or not is a red herring. It has nothing to do with the sale, which without dealers would be robbed of much of its value both to donors and buyers. They help to maintain prices, which achieves one of the objects of the sale. At the sales they are professionals. Outside it, they are men with sympathies to be appealed to like everybody else.

### THE LIABILITY OF A HUSBAND FOR MEDICAL ATTENDANCE ON HIS WIFE.

An interesting case was decided in the High Court of Justice, King's Bench Division, on February 8, on appeal from a decision of a County Court Judge. Briefly, a doctor attended a lady in her confinement, and sent her subsequently his account. She paid part of it, but refused to pay the rest. The doctor sued the lady, whose husband is a member of the Indian Civil Service and at all the material dates was resident in Rangoon. Both the Court of first instance and the Appeal Court decided that the action failed, because the lady was not separated from her husband by anything more than the accident of his profession entailing domicile abroad: that is, was not judicially or by agreement ceasing to live with him. It was therefore held that the doctor in such circumstances has no remedy against the lady herself, but only against her husband. The counsel for the doctor pointed out that if the husband had been sued, the doctor would have been non-suited: so the moral of the case would seem to be that anyone who gives credit to the wife of an Englishman living abroad does so at his own risk and has no remedy whatsoever if his account is not paid. This surprising result may be a correct interpretation of the law, but it seems monstrously unjust from the point of view of common-sense and ordinary equity.

### A LOCAL MEDICAL DIRECTORY.

A publication which reaches us from Southampton appears to break new ground in several directions, as well as to contain the germs of more far-reaching innovations. It is called the Southampton Medical Directory, and its sixty-four pages contain an enormous amount of information which is bound to be useful to the doctors of the Southampton district. It begins with lists of the doctors, dentists, nurses, and midwives resident in the county borough, with addresses, telephone numbers, and other information about their professional activities. Then follows a lot of very useful medico-legal information, especially full when dealing with the medical inspection of school children, maternity and child welfare, notification of infectious diseases, and similar topics of State medicine. After that a full section is devoted to National Insurance matters as they affect the panel practitioner. Much information is given about the various medical societies and committees which exist in the borough, and about the charitable organisations and hospitals which minister to the sick. The editor appeals for additional information whereby future issues may be rendered of enhanced utility. He has certainly deserved well of his colleagues, not only for his novel idea, but also for the amount of hard work he has put into it.

### A HOSPITAL TREASURER ON VOTES FOR WOMEN.

Speeches at annual meetings are all the better for taking a wide view of things, and therefore no one will find fault with Lord Faber for his recent address on being re-elected honorary treasurer of the Harrogate Infirmary. He paid a great tribute to the work which women have done for the nation in every field since the war began, and added: "Some years ago I was associated with the late Lord Cromer in opposing the grant of votes to women. I was wrong. I have changed my mind entirely since the war began, and now I shall do all I can to get women the vote." He added that heavy taxes were crippling among others the county gentleman, who could hardly keep up his estate and house, which could not nowadays be sold, and that this must affect the income of charities. He did not object to the working man getting more, as he was now in munition and other factories. They had also to consider, he said with impressive restraint, what the relative value of gold and paper would be after the war. Lord Faber has left it to us to draw the moral from his remarks, which is, so far as hospital finances are concerned, to realise in time that the backbone of future solvency must be the weekly collection in shop, office, and factory, even among the hospitals of smaller towns and country districts, which have much neglected it in the past. Thus, as we are never tired of repeating, hospital finance must be based on public interest in the life of each institution, which interest must express and organise itself through a variety of subsidiary organisations.

### THE DIFFICULTIES OF FOOD RESTRICTION.

Dr. John Robertson, Medical Officer of Health for Birmingham, has given some interesting news to the *Birmingham Daily Post* on the difficulties of food restriction. He regards the Government scale as liberal, but points out that much depends upon the size of the family and the ages of its members. Since, moreover, persons are at liberty to purchase other articles, each, he points out, may exceed the limit allowed. This, in effect, amounts to an admission that you cannot equalise rations until you have equalised purchasing power. We may recall a remarkable article in the *Morning Post* some weeks ago written by Sir F. Long, formerly Director of Transport and Supplies to the War Office. He said that if the German Government had known what they have since learnt, when introducing complicated food regulations they would never have introduced them, which is another way of putting the question we have here presented. The only solution yet suggested was made, we believe also in the *Morning Post*, by another writer, who proposed that the responsibility should be thrown upon the retailers. People should have the right to choose their own shops, but no shop should be allowed to sell them more than a specified amount of various goods per week, which would be entered on cards that they would have to present at the time of purchase. Such a plan seems possible, though vexatious.

### A HOSPITAL'S MEDICAL STAFF.

All save one member of the medical staff of the Beverley Hospital and Dispensary have resigned. At a subscribers' meeting, presided over by the Mayor and held to arrive at a solution, Mr. C. J. Arden, clerk to the hospital, explained that the cause of the dispute was the liquidation of an overdraft and a balance of £140 which resulted from a special appeal. The medical staff then asked for a rise in their salaries, and in May 1916 propounded a scheme which he said was found unworkable. It involved the payment of £100 per annum. The hospital's directors offered to pay £5 a year, whereupon in June last the medical staff threatened to resign, if the £100 a year they had asked for was not paid. Every medical man in the town was asked by the directors to join the staff, whereupon the old staff said that they would not accept one doctor, Dr. F. H. L. Munro, as a colleague. The directors, added the clerk, had no cause to think him questionable, but all the staff resigned except this gentleman. The Mayor had been empowered to settle the matter, and it should be added that Mr. Arden explained that the directors could not find any complaint against Dr. Munro on professional grounds. For the moment we refrain from comment, until the result of the Mayor's conference with the other doctors is made known.

### WOMEN AS PRESIDENTS OF COTTAGE HOSPITALS

Lady Montagu Turner has been elected to succeed Mrs. Bailey as president of the Romford Victoria Cottage Hospital, which thus possesses a tradition of lady presidents. In the case of the cottage hospitals there is a good deal to commend this plan, if the right type of public-spirited woman is available. No doubt on this score should arise concerning the new president, since she has previously interested herself in hospital work in India, notably in Calcutta and Bombay. The presence of women on committees is often objected to by those who have had no experience of their co-operation. But we may say in the light of this experience that women add to the value of most committees, and often make better chairmen than men, just as they are as a rule more fluent speakers.

### ABBREVIATED ANNUAL REPORTS.

Hospitals and institutions receiving grants from King Edward's Hospital Fund, the Metropolitan Sunday Fund, and the Hospital Saturday Fund have been notified that those bodies will raise no objection during the present period of dear printing and paper if the annual report is reduced to the bare minimum of what is necessary for the information of the subscribers—that is, the list of officers, the report of the executive, and the accounts and statistical tables required by the Funds. That which makes most bulk in the report, the list of subscribers and donors, may be omitted, but must be open to the inspection of anyone entitled to see it at the office of the charity, and, of course, must be examined by the auditors. Opinions are so varied as to the policy and propriety of issuing a full annual report in these times that no doubt many charities will continue to do so. The saving made by the limitation of contents is not so great as may appear at first sight, as a long list of the governors and contributors of an important institution will still remain in type for correction from year to year, for to break up the standing matter and reset it again next year or the year after would be a troublesome and expensive business. The actual issue to the public is another matter, and the saving in printing, postage, and paper is evidently considered by the Central Funds to be of importance.

### A CRISIS AT CHORLTON-ON-MEDLOCK.

The serious financial position of the Chorlton-on-Medlock Dispensary, Manchester, is causing anxiety to the committee, and it was stated at the annual meeting last week that unless more money can be obtained it will be difficult to carry on the work. Over 1,300 patients were treated last year, and though the loss was but £100, this deficiency is a recurring one—hence the crisis. The support of the public does not appear to be extended to dispensaries in anything like the measure it is extended to hospitals. One reason, perhaps, is that dispensaries too often hide their light under a bushel, though why they should do so passes comprehension, for they are of real benefit to poor people, the majority of whom are only just beyond the pale of the Poor Law.

### MILITARY HOSPITAL EXPANSION AT NEWPORT.

Several hundred more beds for sick and wounded soldiers are being provided by the authorities of the 3rd Western General Hospital. At Newport the cavalry depôt has been requisitioned and is being converted into a sectional hospital with accommodation for 300 patients. At Cardiff steps are being taken to obtain an additional 120 beds at the University Settlement. Last week another auxiliary Red Cross hospital was opened by the Lord Mayor at St. Pierre, Newport Road, Cardiff (a commodious residence, housing 43 beds), which has been handed over to the British Red Cross Society for the period of the war by Mr. Max Wideman. Mrs. W. L. Yorath is the commandant of the hospital, which is splendidly equipped.

### WOUNDED ALLIES' RELIEF COMMITTEE.

Concerning last year's work of the Wounded Allies' Relief Committee, 8 Grosvenor Gardens, S.W., some interesting facts are reported. At the Committee's branch of the Kensington War Hospital Supply Depôt, 176 Cromwell Road, South Kensington, no less than 383,500 hospital requisites were made, and from these 170 consignments have been sent to over 120 hospitals. During the year the Committee have sent to the allied countries the following:—To Belgium, motor operating theatre, two bath caravans, two hospital tents, one case of games each to nineteen Belgian hospitals; to France, three motor ambulances, bandages, splints, hospital clothing, and special grants of warm clothing; to Portugal, bandages, splints, hospital clothing; to Russia, four motor ambulances, quantities of splints, bandages, and hospital clothing; to Roumania, drugs, surgical appliances, and hospital requisites to the value of £600; to Serbia (in addition to stores and one motor ambulance sent to the Committee's hospital at Vodena, Greece), one motor-car and large quantities of splints, bandages, and hospital clothing.

### A MUCH-ENDOWED WAR HOSPITAL.

Is there any hospital in any part of the world which can beat the record set up by the Welsh Hospital at Netley? There are 302 beds in the institution, and no fewer than 212—thanks in a great measure to the abounding energy shown by Sir William James Thomas, the honorary treasurer, in gathering in the wherewithal—are named after donors. Thus there are only ninety beds not named after those who maintain them; but even these ninety are likely ere long to join the majority.

### QUEENLY TRIBUTE TO NURSES.

A CHARMING tribute to the good work of health visitors, etc., is paid by Queen Alexandra, who is the patron of the Royal College of St. Katharine. Recently the Poplar Borough Council addressed a letter to Her Majesty expressing gratification at the increasing activities of the Royal College of St. Katharine, and appreciation of the valuable work it is accomplishing in the conservation of infant life in the borough. To this the Council has received from Her Majesty Queen Alexandra a reply in which she rejoices in the fact that it has been found possible to extend the sphere of operations, and sincerely trusts that, with the Council's continued co-operation and support, the all-important and invaluable work of the College in connection with the welfare of infants may steadily increase and prosper. Her Majesty feels that the success which has been achieved by St. Katharine's College is mainly due to the untiring energy and devotion to duty of Miss MacQueen, the Principal, and her lady health visitors, and the unselfish and able help and advice of the medical officer. At the same time the Borough Council sent congratulations to Miss MacQueen, who returns her thanks for the kind expression of the Council's appreciation of the work being done.

### THE RISK OF LIBEL IN MEDICAL PRACTICE.

PART of the playwright's business is to open the eyes of the public to evils which are escaping its attention; and as such a task is never popular at the time, and rarely acknowledged when the evil is remedied, we may be excused for quoting an up-to-date example of dramatic foresight. The Preston Board of Guardians, on the request of another board, has passed a resolution asking the Association of Poor-Law Unions to urge the Government to cancel the existing law which renders a doctor liable to an action for libel if he warns the family of a patient suffering from venereal disease of the nature of the disease and the danger of infection. Other boards will no doubt follow suit, and therefore we may recall that this reform, which was suggested in the Report of the Royal Commission on Venereal Diseases, was urged several years ago by the French dramatist Brieux in "Damaged Goods," a translation of which has been published by Mr. Fifield, but forbidden by the Lord Chamberlain, and also in an English play called "Philip's Wife," by Dr. F. G. Layton. The public which slighted both plays is now acting in accordance with the lesson they taught.

### THE DISCOVERIES OF CONVALESCENCE.

A PHILOSOPHER once expressed his belief that the vast majority of men were square pegs in round holes; that a man became a banker who had a natural genius for keeping sheep, and that a shepherd of his acquaintance, who could neither read nor write, conducted every market day all his transactions in mental arithmetic, and obviously ought to have been a financier. Evidence in support of this contention is provided by the War Exhibition which closed recently at Norwich. It consisted of articles, many of them artistic in aim, which had been made by the wounded in the hospitals of the county. Besides the mere mat and the habitual basket were blouses and embroidery. The blouses had been made by a cowman and a groom, and the stitching, we are assured, was exquisite in detail. We know two former members of religious orders who are adepts at making ladies' hats, but we do not know at present of a man milliner who is a born milker. War and chance make such discoveries rarer than the former, but that the same rule often applies we have no doubt. How many men miss their vocation?

### MEATLESS DAY AT THE BRITISH HOME, STREATHAM.

LONG before Lord Devonport had made his request for voluntary rationing the British Home and Hospital for Incurables at Streatham had instituted a meatless day once in every week. This does not mean that on one day in the week the patients are given fish for dinner, for in the scheme of the Home fish is counted as meat. The *menus* for the meatless day are strong in vegetable dishes, such things as vegetable cutlets and soups are *pièces de résistance*, with macaroni cheese and substantial puddings to follow. We learn that the usual kitchen staff have succeeded in satisfying the patients, who are quite enjoying the novelty, although, no doubt, they go back to their joints on the following day without regret. The chief and generally the only fault in institutional feeding is sameness; there is an almost entire absence of the occasional surprise that one experiences in domestic life, and we can quite understand that the patients at the British Home await the new dishes with interest and appetite. It would be interesting to know if any of our hospitals make full use of batter puddings and suet dumplings in the dietary of patients and staff; each is a dish of a satisfying nature, has excellent food value, and if meat diets are to be reduced their use might be considered.

### THE CAREER OF THE WORKHOUSE CHILD

THE removal of children from the mixed workhouse has been adopted on a sufficiently wide scale to make that old phenomenon the "workhouse child" practically a figure of history. But all is not done, once the Guardians have found suitable homes for them. What happens to them when they grow up? Hitherto they have had to depend upon some individual Guardian or employer to lend them a helping hand at the start, and though, where this is forthcoming, the personal relation is all to the good, it cannot be a reliable stand-by. Thus the after-care of workhouse children may mean much more than some supervision of their convalescence after an illness. The Leeds Board of Guardians have been considering a new scheme in this matter. A committee formed by members of the boarding-out and children's committees is suggested, the object of which would be to find situations for their charges, and supervise their careers till eighteen years of age. Records will be kept, and reports made to the Guardians concerning them. If such records and reports are confidential, the work proposed should be useful and kindly. But though useful it might be also oppressive, prying, and tyrannical. If this can be avoided, the scheme should accomplish much good and set an example worthy to be followed.

### A NEW EDITOR AT BIRMINGHAM.

If the January number of the *Southern Cross* is a trifle late in making its appearance, the new editor shows the right spirit in basing his request for contributions on the plea that "we want a magazine which reflects our spirits and our capacity. We can have it. We must have it." This is tersely and sensibly put. The new number opens with the first part of an article or articles giving the hospital's history since 1908, which is well put together and is interesting reading. The photographs of the Christmas entertainments are not likely to be forgotten. In the true mediæval spirit of Christmas, the Skeleton at the Feast was not left out. There is promise in this number that the new editor is getting to work. If he receives a proper backing of contributions from the staff he will make a good magazine out of their efforts.

### THE "CRAIGLEITH CHRONICLE."

There are, as usual, varied and ambitious verses and articles in the February number of the *Craigleith Hospital Chronicle*. The verses by Helen Neaves, John Hogben, and even the bitter birthday ode to the Kaiser signed "J. H. M. C.," are above the usual magazine standard, and we must also mention the lines by Pte. A. E. Snodgrass, R.F., in the same number on the favourite theme of "Sister." A farce, by the same writer, which was acted by the patients on Boxing Night, is also included. Such attempts are too few and deserve to be encouraged. Perhaps many English people do not know or have forgotten that Scotland observed Sunday, December 31 last, as a day of humiliation and prayer. Sir A. H. L. Fraser, K.C.S.I., took advantage of the occasion to deliver an address on the prayer of Jehoshaphat when he heard that a great multitude was coming up against him. It contains a survey not of the war, but of our feelings at different periods of its course. The hospital notes and the details of Christmas festivities are of local interest but, if we may judge from the opening sentence of Sir Joseph Fayrer's "Foreword," the Colonel is leaving or is not going to be a contributor again.

### A FEAST OF ILLUSTRATIONS.

It has been said that the cry of plagiarism is never raised by first-rate artists, since these know that their store is inexhaustible, and that they can no more be robbed than a spring well. Such a happy assurance must be felt by the artists and the editor of the 3rd London General Hospital's *Gazette*, the illustrations to which are inexhaustible in fun and fancy, and apparently in supply. What could be better than Corporal Kirk's and Private J. H. Dowd's illustrations to the February number, to say nothing of those by Marjory Collins, Lance-Corporal Hodgson Lobley, Ethel Woolmer, and the rest? Among the writers Miss H. M. Nightingale has now made a reputation for herself by contributions in prose and verse which have a directness, simplicity, and humorous observation that sometimes remind one pleasantly of her great namesake. No one who compares these articles with the style of "Notes on Nursing" or "Suggestions for Thought" can fail to see the likeness, and as Florence Nightingale was, at her best, the master of a nervous prose that delighted Mill and Jowett, this is not a small or fantastic compliment to make. We should like to have had a fuller account from the C.O. of his experiences in Mesopotamia, but the truth on any subject is rarely considered printable, and a Royal Commission is sitting.

### THIS WEEK'S DRUG MARKET.

The main event of the week has been an improvement in the demand for quinine and consequently a considerable advance in the price; the bulk of the buying appears to have been done on behalf of an Allied Government; recent experience has been that when large orders of this kind have been satisfied, the price has gone back to something near the level at which it stood before the buying began. An advance in quotations for calomel would not come as a surprise. The value of opium is well maintained. Honey still tends upwards in price, notwithstanding that the value has already been doubled during the past five or six weeks. Citric acid is dearer. In synthetic drugs there are few changes to report; barbitone is still very difficult to obtain and phenazone is becoming scarcer. The scarcity of cocaine is more acute. Bromides are unchanged. The general tendency of prices is in an upward direction, and almost anything which comes from across the sea is likely to continue to advance in value in view of the steady rise in freights and insurance charges.

### TO OUR READERS.

Contributions are specially invited from any of our readers to these columns. They should deal with topical subjects and news. They must be authenticated for the information of the Editor only. The minimum payment if published is 5s. There is no hard-and-fast rule as to space, but notes of about twenty lines in length are preferred.

